# British Medicinal Plants

**Published:** 1890-11

**Authors:** Alfred Heath

**Affiliations:** London, England


### BRITISH MEDICINAL PLANTS.

Alfred Heath, M. D., F. L. S., London, England.

### Ranunculaceæ (Continued).

*Actæa Spicata* (Bane-berry, Herb Christopher).—Found in mountainous woody districts in the North of England. This plant has not been proved, but was mentioned by Ruckert as a remedy in certain cases of neuralgia, characterized by violent tearing and drawing rheumatic pains in *one side* of the face, extending from the teeth of the upper jaw through the malar bone as far as the temple. Contact or movement of the facial muscles produced an excessive aggravation of the pain. The plant is similar in its action to the following American variety in that it affects *one side* of the body. It is very acrid and strongly cathartic; the berries are poisonous and have been fatal to children who have been tempted to eat them. The leaves have been used allopathically as an external application to inflammations, and with much success in tumors of the breast; it is well worth proving, and may equal if not surpass the *Cimicifuga.*

*Actea Racemosa, Cimicifuga Racemosa* (Black Cohosh, Black Snake-root).—This, although not an English plant, is one that grows perfectly in this country, and is so well known to the followers of Hahnemann that I think it advisable to mention it. In the garden it grows to a fine plant, with tall spikes of white flowers; it is perennial, and well worth growing. There is a good proving of this plant. It is especially useful in nervous and rheumatic affections, principally affecting the *left side* of the body; facial neuralgia, pains in the *left side* of the breast, spinal irritation from rheumatic or uterine disorders, neuralgia of the eyeballs, delirium tremens, illusions of vision, hysteria, "nervous sick-headache," vomiting, etc., stiff-neck, lumbago, worse when the patient is standing or sitting still, and in cold and stormy weather; sciatica; articular rheumatism of the lower extremities. The last few years the allopaths have been freely using this remedy, as well as many of our best proven drugs, without acknowledging the source from which they learned their use—*i. e.*, the Homœopathic Materia Medica.

*Pœonia Officinalis* (Rosa Benedicta; common name, Peony).—In heathen mythology called after the physician Pœon, who cured Pluto with it when wounded by Hercules, hence it was held in great esteem by the ancients. This, although not originally an English plant, is one of our commonest garden-flowers, and is so well known to every one that it needs no description. There are several garden varieties of *Pœonia officinalis*, all of them bearing showy and beautifully-colored flowers, and probably producing much the same symptoms when used as drugs. In Homœopathy we use the root of the officinal plant, which comes to us from the South of Europe. It was formerly used as an anodyne. This term is applied to medicines that relieve pain: 1st, either by actually assuaging pain, *paregorics;* 2d, those that relieve by producing sleep, *hypnotics;* 3d, those that give ease by stupefying the senses, *narcotics.* It was also used as a *tonic*, and since the days of Galen the root has been commonly employed as a remedy for epilepsy. For this purpose it was cut into thin slices, which were attached to a string and worn round the neck as an amulet; if this failed to relieve it was given internally, in the form of a powder. Many writers in modern times have declared it to be of no use, or only of use in some cases. The reason of this is quite evident; there can be no specific in any disease, and if in the "old school" they had a law to guide them, they would be able, as in Homœopathy, to select the drug that had an affinity of symptoms (as shown by its effect on the healthy) to the individual under treatment. First, on healthy people the *Pœnia produces*, among other things, many varieties of pain, headaches, boring, darting, tearing, gnawing, sticking with pressure, aching (*Paregoric*), rushes of blood to the head, restless sleep (*Hypnotic*), with fancies and dreams. Second, languor, weariness on walking, heaviness in the chest and limbs in the open air, languor and heaviness of the limbs relieved after eating, great prostration in the evening, hence, its power to relieve as a tonic. Third, it produces many symptoms similar to epilepsy—rush of blood to the head, nausea, hissing in the head, vanishing of the senses (*Narcotic*), fainting after walking up-hill, vertigo during every motion, constant reeling sensation in the head, staggering to and fro, heat in the head, "*crawling in the fore-arm as from something alive, transitory creeping in the fingers and sides.*" Now, in epilepsy, one of the most striking premonitory symptoms is what is called the *Aura epileptica*, a sensation compared to a stream of warm or cold air, or the trickling of water, *or to the creeping of an insect*, which commences at the extremity of a limb and gradually runs along the skin toward the head. With *Pœonia officinalis* I come to the end of, and very reluctantly close, the magnificent natural order *Ranunculaceæ*, an order containing so many splendid medicines, and deservedly occupying the position of Natural Order No. 1.

